# Rupture of posterior cruciate ligament leads to radial displacement of the medial meniscus

**DOI:** 10.1186/s12891-017-1646-6

**Published:** 2017-07-11

**Authors:** Can Zhang, Zhenhan Deng, Wei Luo, Wenfeng Xiao, Yihe Hu, Zhan Liao, Kanghua Li, Hongbo He

**Affiliations:** Department of Orthopaedics, Xiangya Hospital, Central South University, No.87 Xiangya Road, Changsha, Hunan 410008 China

**Keywords:** PCL rupture, Medial meniscus, Radial displacement

## Abstract

**Background:**

To explore the association between the rupture of posterior cruciate ligament (PCL) and the radial displacement of medial meniscus under the conditions of different flexion and various axial loads.

**Methods:**

The radial displacement value of medial meniscus was measured for the specimens of normal adult knee joints, including 12 intact PCLs, 6 ruptures of the anterolateral bundle (ALB), 6 ruptures of the postmedial bundle (PMB), and 12 complete ruptures. The measurement was conducted at 0°, 30°, 60°, and 90° of knee flexion angles under 200 N, 400 N, 600 N, 800 N and 1000 N of axial loads respectively.

**Results:**

The displacement values of medial meniscus of the ALB rupture group increased at 0° flexion under 800 N and 1000 N, and at 30°, 60° and 90° flexion under all loads in comparison with the PCL intact group. The displacement values of the PMB rupture group was higher at 0° and 90° flexion under all loads, and at 30° and 60° flexion under 800 N and 1000 N loads. The displacement of the PCL complete rupture group increased at all flexion angles under all loads.

**Conclusions:**

Either partial or complete rupture of the PCL can increase in the radial displacement of the medial meniscus, which may explain the degenerative changes that occuring in the medial meniscus due to PCL injury. Therefore, early reestablishment of the PCL is necessarily required in order to maintain stability of the knee joint after PCL injury.

## Background

The menisci are two crescent-shaped fibrocartilage structures located on the joint surface of the medial and lateral tibial plateau. The triangular cross sections of the menisci, which are slightly concave above and flat below, form a deep depression on the tibial plateau so that the ball-shaped femur condyle can be well accommodated [1]. The meniscus plays an important role in the mechanisms of load bearing, load transmission and shock absorption, and is also crucial to the lubrication and nutrition of articular cartilage [2]. Displacement of the meniscus to the periphery radially is referred to as “radial displacement of the meniscus” or “meniscal subluxation” or “meniscal extrusion” This phenomenon is commonly observed through magnetic resonance imaging (MRI) and ultrasound examinations [3–5]. Radial displacement may be linked to a reduction in meniscal function and other disorders of the knee. Recently, researches have been done to study the radial displacement of the medial meniscus [6–10], the incidence of which increases with osteoarthritis (OA) progression. Radial displacement of the medial meniscus also occurs as a complication upon the completion of meniscal transplantation [9, 10]. However, the association between the rupture of posterior cruciate ligament (PCL) rupture and the meniscus displacement has not been clearly elaborated.

The PCL is an important structure that helps to ensure maintain the stability of the knee joint and to prevent the posterior displacement of the tibia [11]. A number of studies have been conducted to examine the effects of PCL injury on the biomechanics of the knee joint [12, 13], but the clinical and biomechanical researches that had been launched on the relationship between PCL injuries and the menisci was very limited [14–16]. We have performed a cadaveric study previously to investigate the biomechanics of how partial and complete PCL ruptures affect the lateral meniscus displacement at various flexion angles under different axial loads, and found that even partial PCL rupture could initiate lateral meniscus displacement, which became more obvious at larger flexion angles and greater loads. [17]. As part of our PCL and meniscus research series, a biomechanical testing system was applied to record the radial displacement of the medial meniscus under three different conditions, namely the anterolateral bundle (ALB) rupture, the posteromedial bundle (PMB) rupture and the PCL complete rupture. The objective of the present study was to examine the impact of PCL rupture on the radial displacement of the medial meniscus by biomechanical testing, for various flexion angles and different axial loads.

## Methods

### Subjects

The experiment of the present study had obtained approval from the Medical Ethics Committee of Xiangya Hospital, Central South University (Grant number: 201,212,062). As described previously [17], a total of 12 fresh human knees were collected from 6 human cadavers (average age of subject: 30.6 years, ranging from 25 to 38 years) and used as specimens. The deaths of the subjects were caused by accidents or other reasons that did not affect the normal structure and function of the knee. The donors’ family had been informed of the objective of the present research and had signed a consent form for the relevant experiment and publication. Macroscopic inspections and radiological examinations were performed to rule out fractures, tumors, severe osteoporosis, degenerative joint disease and other anomalies. The posterior drawer test was used to exclude specimens with PCL damage. Each whole knee was cut off with a section of 30 cm reserved for both the femoral and tibial side, keeping the skin and soft tissue intact. The specimens were covered by the sterile gauze soaked in normal saline (NS) and then sealed in double plastic bags. All the specimens were stored at −70 °C, and the length of the low-temperature storage time was no more than 3 months.

### Group of experiments and test procedures

As described previously [17], the specimens were grouped into four sets following the sequence in which the experiments were conducted: the PCL intact group (*n* = 12), the ALB rupture group (*n* = 6), the PMB rupture group (*n* = 6) and thePCL complete rupture group (*n* = 12). The sample size was calculated using an online sample size calculator which indicated that the sample size was suitable to detect a difference between treatments.

The specimens were thawed before conducting the experiments, and the soft tissues at the end of the femur and tibia were removed, leaving ones around the knee joint intact. Then, both the ends of the femur and tibia were fixed in a cylinder, ensuring that the specimens were firmly settled during the test. Subsequently, the cadaveric knees were fixed onto the cylindrical clamps of a universal testing machine (CSS-88100, ChangChun, China), with the femur side fixed on the top and the tibia side at the bottom. During the testing process, the knee was in extension and postured with a 10° valgus angle from the vertical axis, which was to simulate the 10 ° valgus angle of a normal knee. The quadriceps was fixed to the femur using wires with a tension of 100 N. The posterior articular capsule was cut through a posterior midline incision in order to expose but not to cut off the PCL. The middle point of the medial margin of the medial meniscus was exposed through a medial longitudinal incision. After removing the surrounding soft tissues and the those in the medial margin of the tibial plateau, a 200 N load was imposed to the specimens with the speed of 0.5 mm/s. This procedure was repeated 20 times to eliminate the innate viscosity of the specimens.

**Fig. 1:**
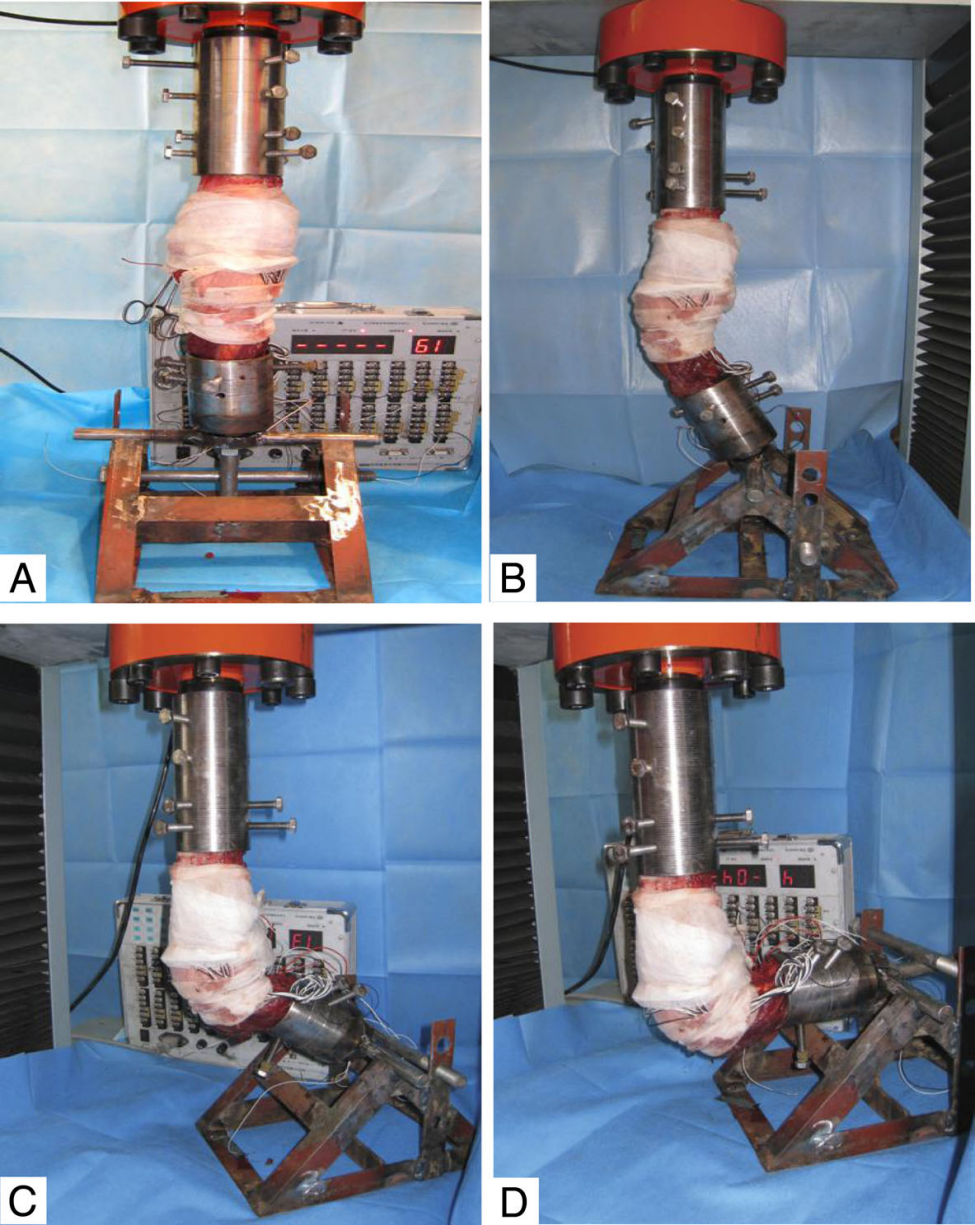
Fixation of knee specimens for biomechanical testing at 0° (**a**), 30° (**b**), 60° (**c**) and 90°(**d**) of flexion

Balance calibration was performed to the static strain measuring device before the testing. Then experiments were conducted at 0°, 30°, 60° and 90° of flexion respectively for the specimens (Fig. 1). A continuously-increasing axial load (0–1000 N) was imposed with the speed of 0.5 mm/s. The elasticity of the specimens was restored at 10-min intervals. The radial displacement of the medial meniscus was defined as the distance from the midpoint of the peripheral margin of the medial meniscus to the midpoint of the edge of the medial tibial plateau. This distance was measured by a digital caliper under 200 N, 400 N, 600 N, 800 N and 1000 N loads respectively. After that, a static strain measuring device was engaged to record the static strain in every channel under the desired conditions. When the aforementioned procedure was complete, the specimens were randomly divided into the ALB rupture group (*n* = 6, where the ALB was transected) and the PMB rupture group (*n* = 6, where the PMB was transected). The test procedure described above was repeated for these two groups. Lastly, the PCL was transected for all the 12 specimens in order to create the PCL complete rupture group, and the same test procedure was performed. During the whole experiment, the specimens were maintained at an appropriate and stable level of humidity and temperature.

### Statistical analysis

All the data are available in the text. The software SPSS 16.0 (version 15.0 for Windows; SPSS Inc., Chicago, IL, USA) was applied for statistical analysis and management. The data were expressed as the mean ± SD. The one-way analysis of variance (ANOVA), SNK-q and Dunnett’s T3 were applied respectively multi-sample means comparisons, heterogeneity of variance. Any difference with *P* < 0.05 was considered as statistically significant.

## Results

Table 1 presents the radial displacements of the medial meniscus of all groups at different knee flexion angles under the various loading conditions. When compared with the PCL intact group, the displacement of the ALB rupture group was higher under the load of 800 N and 1000 N (*P* < 0.05). In the PMB rupture and PCL complete rupture groups, the displacement was higher under all loads (*P* < 0.05). In comparison with the with the ALB rupture group, the displacement increased significantly in the PMB rupture and PCL rupture groups under all loads (*P* < 0.05). However, in comparison with the PMB rupture group, the displacement of the PCL complete rupture group increased significantly under the load of 200 N, 400 N and 600 N (*P* < 0.05), but whereas showed no significant increases under the load of 800 N and 1000 N (*P* > 0.05).

**Table 1:** The radial displacement of medial meniscus at different flexion angles under various loads for all groups (means & SD, mm)

Flexion angels	Groups	Loading conditions				
		200 N	400 N	600 N	800 N	1000 N
0°	PCL intact group	1.18(0.12)	1.19(0.14)	1.26(0.19)	1.41(0.19)	1.57(0.20)
	ALB rupture group	1.22(0.18)	1.31(0.11)	1.47(0.19)	2.09(0.18)^a^	2.29(0.18)^a^
	PMB rupture group	2.05(0.13)^a,b^	2.27(0.11)^a,b^	2.81(0.11)^a,b^	3.36(0.12)^a,b^	3.57(0.17)^a,b^
	PCL rupture group	2.16(0.18)^a,b^	2.35(0.22)^a,b^	3.06(0.41)^a,b^	3.44(0.40)^a,b,c^	3.89(0.20)^a,b,c^
30°	PCL intact group	1.23(0.15)	1.37(0.17)	1.44(0.18)	1.61(0.16)	1.77(0.16)
	ALB rupture group	3.24(0.21)^a^	3.63(0.25)^a^	3.96(0.19)^a^	4.06(0.36)^a^	4.16(0.19)^a▲^
	PMB rupture group	1.25(0.19)^b^	1.41(0.18)^b^	1.56(0.14)^b^	2.10(0.19)^a,b^	2.31(0.29)^a▲,b▲^
	PCL rupture group	3.40(0.24)^a,c^	3.76(0.19)^a,c^	4.16(0.17)^a,c^	4.55(0.22)^a,b,c^	4.98(0.15)^a▲,b▲,c▲^
60°	PCL intact group	1.26(0.15)	1.34(0.12)	1.53(0.16)	1.68(0.13)	1.86(0.12)
	ALB rupture group	3.72(0.26)^a^	4.14(0.22)^a▲^	4.41(0.17)^a^	4.64(0.24)^a^	5.09(0.21)^a^
	PMB rupture group	1.36(0.27)^b^	1.47(0.12)^b▲^	1.60(0.19)^b^	2.36(0.21)^a,b^	2.59(0.19)^a,b^
	PCL rupture group	3.95(0.23)^a,c^	4.26(0.16)^a▲,c▲^	4.59(0.22)^a,c^	5.11(0.30)^a,b,c^	5.87(0.24)^a,b,c^
90°	PCL intact group	1.12(0.11)	1.40(0.16)	1.66(0.16)	1.76(0.16)	2.02(0.16)
	ALB rupture group	3.60(0.16)^a▲^	4.03(0.16)^a^	4.23(0.22)^a^	4.49(0.20)^a^	4.92(0.20)^a^
	PMB rupture group	2.21(0.28)^a▲,b▲^	2.42(0.28)^a,b^	3.06(0.28)^a,b^	3.64(0.28)^a,b^	4.08(0.29)^a,b^
	PCL rupture group	4.64(0.24)^a▲,b▲,c▲^	4.98(0.24)^a,b,c^	5.21(0.25)^a,b,c^	5.47(0.22)^a,b,c^	5.97(0.15)^a,b,c^

*PCL* posterior cruciate ligament, *ALB* anterolateral band, *PMB* posteromedial band

^▲^Dunnett-T3 test

^a^ *P* < 0.05 compared with PCL intact

^b^ *P* < 0.05 compared with ALB rupture

^c^ *P* < 0.05 compared with PMB rupture

At 30° of flexion, the displacement of the ALB rupture group and the PCL complete rupture group was significantly higher than that of the PCL intact group under all loads (*P* < 0.05). In the PMB rupture group, the displacement increased markedly under the load of 800 N and 1000 N (*P* < 0.05), but showed no significant elevation under other loading conditions (*P* > 0.05). The displacement of the PMB rupture group differed significantly from that of the ALB rupture group under all loads, and the displacement of the PCL complete rupture group differed significantly from the ALB rupture group under the load of 800 N and 1000 N (*P* < 0.05). In comparison with the PMB rupture group, the displacement of the PCL complete rupture group increased significantly under all loads (*P* < 0.05).

The results at 60° of flexion were similar to 30°. In comparison with the PCL intact group, the displacement of the ALB rupture and PCL complete rupture groups was higher under all loads (*P* < 0.05). The displacement of the PMB rupture group higher under the load of 800 N and 1000 N (*P* < 0.05). The displacement of the PMB rupture group differed significantly from that of the ALB rupture group under all loads, and the displacement of the PCL rupture group differed significantly from the ALB rupture group under the load of 800 N and 1000 N (*P* < 0.05). In comparison with the PMB rupture group, the displacement showed a significant increase in the PCL complete rupture under all loads (*P* < 0.05).

At 90° of flexion, mutual comparisons of the displacement among the various groups revealed statistically significant differences under all loads (*P* < 0.05). The correlation of the displacement of the medical meniscus in each group increased in the following sequence: PCL complete rupture group > ALB rupture group > PMB rupture group > PCL intact group.

## Discussion

The menisci are indispensable structures of the knee that protect the articular surfaces of the knee from axial loads [18]. Boxheimer demonstrated that most obvious medial meniscus movement is detected in the anterior horn when the knee in supine and externally rotated at 90° flexion position, through monitoring meniscal movement in asymptomatic knees via a MRI scanner [19]. Meniscal subluxation is generally defined as the distance between the peripheral border of the meniscus and the edge of the tibial plateau over 3.0 mm [20], and this criterion was applied in the present study. It was found that the medial meniscus displacement increased significantly increased along with the change of loads and flexion angles. An intact PCL restricted meniscal movement such that medial meniscus displacement fluctuated between 1.0 mm and 2.0 mm, which falls within the range of physiological movement (<3.0 mm). Although the displacement did not vary greatly with the change of loads and flexion angles when the PCL was intact, it increased significantly under the conditions in which the PCL was completely or even partially ruptured.

The results suggested that the medial meniscus radial displacement at 0° of knee flexion after ALB rupture (1.25-2.31 mm) remained a physiological movement, while it increased significantly after PMB rupture (≥3.0 mm), indicating instability of the knee joint, or even subluxation. It was inferred that ALB is secondary to PMB in maintaining the stability at 0° of flexion. When the knee was flexed to 30° and 60° under a load less than 600 N, no significant difference in displacement was observed between the PMB rupture and PCL intact groups. Therefore, the displacement in both groups was still considered physiological movement. However, the displacement (>3.0 mm) occurred in the ALB rupture and PCL complete rupture groups under the same load and flexion angle. Such results indicated that PMB is secondary to ALB in maintaining the stability at 30° and 60° of flexion. However, no complete linear relationship between loading and medial meniscus displacement was found when either the ALB or the PMB was ruptured. An apparent slope change was noted in the displacement curve when the load was over 600 N, suggesting that fiber bundles had the ability of self-adjustment within a certain range of loads when the PCL was incompletely ruptured. This compensatory mechanism redistributed stress and reduced the displacement of the meniscus. However, when the load increased beyond a threshold value, the meniscus could cause obvious signs of knee joint instability or even subluxation. When flexed to 90°, the meniscus displacement differed significantly within the intra-group comparisons of all groups (*P* < 0.05). Subluxation occurred at 600 N and above in the PMB rupture group, and at 200 N and above in the ALB rupture and PCL complete rupture groups. Therefore, both ALB and PMB, especially the former, are associated with knee joint stability at 90° of flexion.

As the normal position of the meniscus is a key factor in load bearing and transmission, abnormal menisci are a critical cause of articular cartilage degeneration, and can result in the progression of OA in the knee [21]. Meniscus subluxation exerts the similar effect to a partial or complete resection of the meniscus, which can reduce the contact area of the tibial-femoral joint, increase the joint strain and accelerate the development of OA [22]. Kenny found that knee joints with radial displacement of the medial meniscus observed from MRI were also have a radiographic Fairbank’s sign, a feature of meniscectomy-induced OA, evident in X-rays [3]. Meniscal subluxation has been previously proved to be a cause of joint space narrowing [23, 24], leading to the conclusion that early knee OA may be attributed to meniscal subluxation, instead of the thinning of the articular cartilage [25]. Sugita observed well-preserved medial menisci in cases of severe varus OA patients, and inferred that the menisci were saved from radial displacement, which precedes the narrowing of the medial joint space and leads to the progression of varus OA [26]. Roubille found more loss of cartilage volume in the lateral plateau in patients with meniscal subluxation. This finding indicated that the meniscal extrusion in the medial compartment can not only cause loss of cartilage volume in the ipsilateral compartment, and affect the contralateral side; meanwhile, it may also serve as a trigger of pathways causing cartilage damage throughout the whole joint [21]. PCL rupture may result in abnormal strain and disrupt the normal creep kinetics of the meniscus, leading to a series of physical problems, including the malnutrition of the meniscal tissue, the degeneration of the meniscus, the loss of hoop stress resistance, the meniscus extrusion, the decrease in load-bearing ability, the knee joint instability, articular cartilage damage and OA [18]. On the whole, meniscal subluxation may serve as a stimulating factor during the development of OA. A stable knee joint can effectively prevent meniscus subluxation and articular cartilage degeneration.

The present study is subject to several limitations. First, the study had a relatively small sample size. In order to draw more definitive conclusions, larger-scale investigations needs to be carried out. Second, the specimens were collected from young donors who might not be able to represent the status of all population especially the senior people. Third, it is impossible to simulate complex loading conditions such as tibial rotation and mediolateral forces in cadaveric knees. Fourth, the same knees were used during sequential testing, which may lead to fatigue failure of the surrounding secondary restraints. Therefore, 10 min interval was set during the testing to restore to the elasticity of the specimens. Finally, the dissection of the articular capsule and the adjacent soft tissue may change the normal physiological structure, which leads to errors in measurement. Thus a longitudinal incision was adopted to minimize the effect on the knee joint stability.

## Conclusions

The present study indicates that either partial or complete rupture of the PCL can increase the radial displacement of the medial meniscus, which may explain the degenerative changes occuring in the medial meniscus upon PCL injury. Therefore, early reestablishment of the PCL is necessary for maintaining the stability of the knee joint after PCL injury.

## Abbreviations

ALB: Anterolateral bundle
MRI: Magnetic resonance imaging
NS: Normal saline
OA: Osteoarthritis
PCL: Posterior cruciate ligament rupture
PMB: Posteromedial bundle
SNK-q test: Student-Newman-Keuls test
